# A tale of topoisomerases and the knotty genetic material in the backdrop of *Plasmodium* biology

**DOI:** 10.1042/BSR20212847

**Published:** 2022-06-14

**Authors:** Priyanka Singh, Khushboo Rani, Akanksha Gotmare, Sunanda Bhattacharyya

**Affiliations:** Department of Biotechnology and Bioinformatics, School of Life Sciences, University of Hyderabad, Hyderabad, India

**Keywords:** malaria, Plasmodium falciparum, topoisomerases

## Abstract

The untangling or overwinding of genetic material is an inevitable part of DNA replication, repair, recombination, and transcription. Topoisomerases belong to a conserved enzyme family that amends DNA topology during various processes of DNA metabolism. To relax the genetic material, topoisomerases transiently break the phosphodiester bond on one or both DNA strands and remain associated with the cleavage site by forming a covalent enzyme–DNA intermediate. This releases torsional stress and allows the broken DNA to be re-ligated by the enzyme. The biological function of topoisomerases ranges from the separation of sister chromatids following DNA replication to the aiding of chromosome condensation and segregation during mitosis. Topoisomerases are also actively involved in meiotic recombination. The unicellular apicomplexan parasite, *Plasmodium falciparum*, harbors different topoisomerase subtypes, some of which have substantially different sequences and functions from their human counterparts. This review highlights the biological function of each identified *Plasmodium* topoisomerase along with a comparative analysis of their orthologs in human or other model organisms. There is also a focus on recent advancements towards the development of topoisomerase chemical inhibitors, underscoring the druggability of unique topoisomerase subunits that are absent in humans. *Plasmodium* harbors three distinct genomes in the nucleus, apicoplast, and mitochondria, respectively, and undergoes non-canonical cell division during the schizont stage of development. This review emphasizes the specific developmental stages of *Plasmodium* on which future topoisomerase research should focus.

## Introduction

Malaria is a life-threatening infectious disease caused by the blood-borne protozoan parasite, *Plasmodium*. The World Health Organization (WHO)’s 2021 world malaria report indicated that there were 241 million malaria cases and 627,000 deaths in 2020, which is an increase of 69,000 deaths from the previous year [1]. Of the five *Plasmodium* species that are known to infect humans, *P. falciparum* is the most prevalent and deadliest, resulting in more than 90% of malaria-related deaths worldwide. The pathogenicity and virulence of this parasite can be imputed to its complex two-host life cycle. The malaria parasite undergoes a series of asexual replications that include the formation of gametocytes in an intermediate vertebrate host followed by gamete maturation and meiosis in the definitive insect host [2]. The eukaryote cell cycle is well defined with successive phases involved in DNA replication (S-phase) and cell division (M-phase) along with two gaps (G_1_ and G_2_ phases) which are strictly governed by cell cycle regulators [3]. However, the *Plasmodium* cell cycle diverges substantially from the classic cell cycle, undergoing a process called schizogony, in which a large number of daughter nuclei are produced within a common cytoplasm [4–6]. This type of endoreduplication requires several rounds of nuclear, mitochondrial, and apicoplast genome replication in the absence of cytokinesis followed by intricate mechanisms of segregation and condensation with complex checkpoints that remain poorly characterized. This non-canonical mode of DNA replication and cell division occurs at three different times, in the liver, in the blood cells of the vertebrate host, and during oocyst formation in the midgut of the insect host.

DNA replication and other cellular processes require entry into DNA during which the double-helical structure is temporarily broken and resealed. This essential activity is accomplished by a group of enzymes called DNA topoisomerases [7]. During DNA replication, the two intertwined strands are separated and used as a template for the synthesis of new daughter strands. This process creates positive supercoils from DNA overwinding ahead of the replication fork as well as entwining of the daughter strands, generating precatenanes [8]. These topological perturbations in the supercoiled double-helical structure of DNA are resolved by topoisomerases to prevent the replication fork from stalling and to ensure that genome segregation during cell division is not impeded by linking of the daughter strands [9].

Given their indispensable role in resolving topological DNA, topoisomerases are characterized as promising drug targets to treat a variety of bacterial infectious diseases such as pneumonia, tuberculosis, and salmonellosis [10–12]. Topoisomerases such as Topo II have also been targeted in the treatment of chronic diseases like cancer [13], and have been explored as novel targets for malaria. DNA topoisomerases from unicellular pathogens like *Plasmodium* exhibit significant sequence variation from their human counterparts. Additionally, the orthologues of some of the parasitic topoisomerases are absent in mammalian cells. The discrete properties of malaria topoisomerases provide new options for drug targeting that help to address the increase in drug resistance.

Some recent reviews have focused on in-depth structural information about topoisomerase subtypes [14,15] from bacteria and protozoan parasites. This review focuses instead on recent findings on the biological function of various topoisomerases in the malaria parasite and gives a comparative analysis of their respective homologs in other model organisms. Two Type I and six Type II topoisomerases have been annotated in the *P. falciparum* genome (Table 1). The putative homologs of each topoisomerase have been identified in other apicomplexan parasites (Table 2), and human, yeast, and bacterial homologs of *P. falciparum* topoisomerases have also been presented (Table 3). This review presents a composite figure that compare the amino acid sequence of each topoisomerase with its human ortholog using dot plot analysis (Supplementary Figure S1). Findings from drug-inhibitor analysis of each *Plasmodium* topoisomerase (Table 4) and potential applications are also shared.

**Table 1:** *Plasmodium falciparum* topoisomerase classification and localization

Type	Locus	Protein	Protein description	Localization	Reference
IA	PF3D7_1347100	XP_001350185.1	Topo III	Nucleus and mitochondria	[29]
IB	PF3D7_0510500	XP_001351663.1	Topo IB	Not determined	-
IIA	PF3D7_1433500	XP_001348490.1	Topo II	Nucleus	[63]
IIA	PF3D7_1223300	XP_001350630.1	Gyrase A	Apicoplast	[67]
IIA	PF3D7_1239500	XP_001350789.1	Gyrase B	Apicoplast	[67]
IIB	PF3D7_1365600	XP_001350366.1	Topo VIB	Nucleus and organelle fraction	[72]
IIB	PF3D7_1217100	XP_001350573.2	Spo11	Not determined	-
IIB	PF3D7_1027600	XP_002585415.1	Spo11, putative	Not determined	-

**Table 2:** Apicomplexan protein homologs of *P. falciparum* topoisomerases

Type	Protein	Accession numbers of apicomplexan homologs identified						
		*P. berghei*	*P. vivax*	*P. yoelii*	*P. chabaudi*	*C. parvum*	*C. hominis*	*T. annulata*
IA	XP_001350185.1	XP_034423756.1	XP_001614250.1	XP_728858.2	XP_016654744.1	XP_001388262.1	XP_668452.1	XP_955385.1
IB	XP_001351663.1	XP_034422310.1	XP_001613277.1	XP_725660.1	XP_740287.2	XP_628499.1	XP_668245.1	XP_952523.1
IIA	XP_001348490.1	XP_034421913.1	XP_001616638.1	XP_022813283.1	XP_016655535.1	XP_625680.1	XP_665482.1	XP_952252.1
IIA	XP_001350630.1	XP_034424206.1	XP_001617321.1	XP_728165.3	XP_740732.2	-	-	-
IIA	XP_001350789.1	XP_034424367.1	XP_001613931.1	XP_724272.1	XP_744228.2	-	-	-
IIB	XP_001350366.1	XP_034422620.1	SCO67967.1	XP_022812488.1	VTZ69403.1	-	-	-
IIB	XP_001350573.2	XP_034424152.1	VUZ98882.1	XP_022812940.1	VTZ71025.1	QOY39886	PPS95409.1	-

**Table 3:** Human, yeast, and bacterial homologs of *P. falciparum* topoisomerases

Type	Locus	Protein	Human		*Saccharomyces cerevisiae*		*Escherichia coli*	
			Name	Accession number	Name	Accession number	Name	Accession number
IA	PF3D7_1347100	XP_001350185.1	TopoIIIα	NM_004618.5	Top3	NM_001182121.1	TopoIII	946141 (TopB)
IB	PF3D7_0510500	XP_001351663.1	Top1	NM_003286	Top1	NM_001183260.1	ABSENT	
IIA	PF3D7_1433500	XP_001348490.1	TopoIIα	NM_001067.4 (Top2A)	Top2	M13814.1	ABSENT	
IIA	PF3D7_1223300	XP_001350630.1	ABSENT		ABSENT		GyrA	946614
IIA	PF3D7_1239500	XP_001350789.1	ABSENT		ABSENT		GyrB	948211
IIB	PF3D7_1365600	XP_001350366.1	Topo6BL	NM_024650.3	ABSENT		ABSENT	
IIB	PF3D7_1217100	XP_001350573.2	Spo11	NM_012444.3	Spo11	NM_001179102.1	ABSENT	

**Table 4:** Inhibitors targeting *Plasmodium* topoisomerases

S. No.	Name of the inhibitor	Target topoisomerase of *P. falciparum*	Mode of action of the inhibitor/biological effect	References
1.	Camptothecin (CPT)	Topo IB	Inhibits the *in vivo* nucleic acid biosynthesis of the parasite; Inhibits the super-coiled plasmid DNA relaxation activity	[74,36]
2.	N-tosyl-azapterocarpan (LQB223)	Topo IB	Inhibits intra-erythrocytic growth of *P. falciparum;* Inhibits *P. berghei* infection in mice	[75]
3.	Synthetic peptide WRWYCRCK	Topo IB	Inhibits the super-coiled plasmid DNA relaxation activity and DNA cleavage activity of TopoIB	[76]
4.	Etoposide	Topo II, Topo VI	Inhibits the growth of the asexual stage of the parasite by causing double strand break at the chromosomal DNA; Inhibits the decatenation activity of TopoVI	[64,77,72]
5.	Ciprofloxacin	Topo II, Gyrase	Inhibits the decatenation activity of purified TopoII; Causes cleavage of the apicoplast genome and exerts delayed death to the parasites	[64,79]
6.	GSK299423	Topo II	Inhibition results in generation of asymmetric single-stranded break in the plasmid DNA; Inhibits the proliferation of parasites within erythrocytes	[64]
7.	3,6-diamino-1'- amino-9-anilinoacridine	Topo II	Inhibits the decatenation activity of TopoII and inhibits parasite growth within the erythrocytes	[78]
8.	Novobiocin	Gyrase	Inhibits the ATPase activity of the enzyme; Inhibits the trophozoite to schizont stage transition of the parasite and specifically reduces the apicoplast genome content	[69]
9.	Purpurogallin (PPG)	Gyrase	Inhibits the DNA binding activity of GyrB and inhibits the growth of blood stage parasites	[81]
10.	Radicicol	Topo VIB	Inhibits the decatenation activity of TopoVI; Inhibits the schizont to ring transition of the parasite in a reversible manner during intra-erythrocytic development of the parasite; It also reduces the mitochondrial genome content of the parasite	[72,84]

## Type I DNA topoisomerase

### Prelude

Type I DNA topoisomerases are monomeric enzymes and do not require ATP to function. These enzymes are divided into three subgroups based on their structure and mode of action: A, B, and C. The A family topoisomerases, Topo IA and Topo III, remain transiently associated with the 5′ phosphoryl group and require divalent cations for DNA binding. In contrast, the B and C family enzymes [16,17], Topo IB and Topo V, respectively, remain associated with the 3′ phosphoryl group and do not require divalent cations for DNA binding. There is a distinct difference in the strand passage activity of these subtypes of enzymes which delineates their role in DNA replication. While the Topo IA enzyme binds to the broken DNA forming a DNA-gate through which another DNA strand can pass, the Topo IB enzyme rotates the nicked DNA strand relative to the other strand. Type IA topoisomerases can only relax the negative DNA supercoils but Type IB and Type IC can relax both positive and negative supercoils [7]. A study in the budding yeast, *Saccharomyces cerevisiae*, showed that positive supercoils created during the progression of a replication fork, are efficiently removed by Type IB [18] but not Type IA (Topo III) topoisomerases. However, once various aberrant structures are generated during replication fork progression, they can no longer be resolved by Type IB topoisomerases. These forms, known as chicken foot structures, resemble Holliday Junctions (HJ) and can be resolved by Topo III along with RecQ helicases (BLM helicase) and RMI (RecQ mediated genome instability) [19]. These types of HJ intermediates are also generated during recombination at the telomere ends [20] and during homologous recombination (HR)-mediated DNA double-strand break repair. TopoIII–BLM–RMI complexes catalyse the decatenation of such intermediates and can together reduce sister chromatid exchange (SCE) [21,22] during HR, thereby maintaining genome integrity. In humans, Topo IIIα is required for the decatenation of hemicatenane structures generated at the termination of human mitochondrial DNA replication, and the loss of Topo IIIα impairs mitochondrial genome segregation [23]. TopoIIIβ is a sole RNA topoisomerase that binds to a specific group of mRNAs to relieve topological stress during transcription. The majority of these transcripts are translated into proteins involved in synapse structure and activity. Indeed, the absence of Topo IIIβ in mice is shown to cause cognitive defects and psychiatric disorders [24,25].

While most Topo IB enzymes are only present in eukaryotes, one has been identified in the pathogenic bacterium, *P. aeruginosa* [26]. In addition to releasing DNA supercoils by acting as a swivel during DNA replication and transcription [27], Topo I enzymes serve as an integral part of the transcription machinery by remaining associated with RNA polymerase II (RNAPII) in the proximity of the transcription start site (TSS) and the transcription termination site (TTS) of the actively transcribing genes [28].

**Figure 1:**
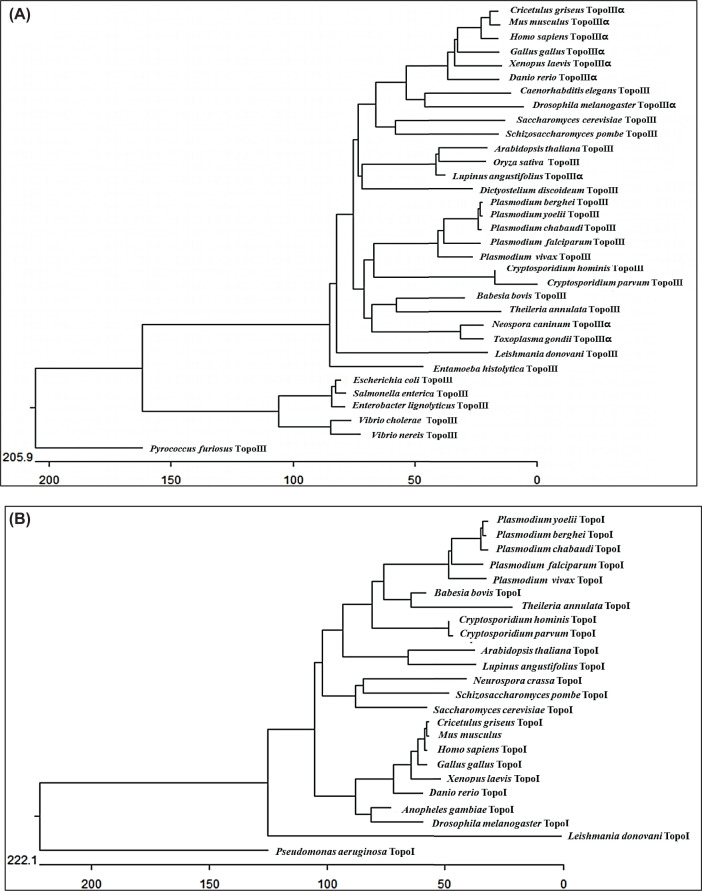
Molecular phylogenetic analysis of type I topoisomerases (**A**) Phylogenetic tree was constructed using amino acid sequences of TopoIII from apicomplexan parasites, higher eukaryotes and bacterial species; (**B**) Similar to (A), it was constructed using amino acids sequences of TopoIB from human and its orthologs from other organisms as presented. Phylogenetic tree was generated by performing Multiple Sequence Alignment using ClustalW available in DNASTAR's MegAlign Software. The tree is typically drawn to scale, with branch lengths measured in number of amino acid substitutions per 100 residues, shown on *X*-axis.

The *Plasmodium* genome includes Topoisomerase III and Topoisomerase IB from the Type I family. Phylogenetic analysis showed that during evolution Topoisomerase III and Topo IB of *P. falciparum* remained closely related to their orthologs in other *Plasmodium* species and distinct from those in yeast and higher eukaryotes (Figure 1A,B). The accession numbers of Topo III and Topo IB from various organisms used in our analysis are presented in the Supplementary Data.

### Role of topoisomerase III in *Plasmodium* biology

*P. falciparum* Topoisomerase III (Gene ID: PF3D7_1347100) encodes a 710 amino acid protein. There are some differences between the amino acid sequences of PfTopo III with the hTop IIIα, and the full-length enzyme shares 39% sequence identity with its human orthologue. While the TOPRIM domain and catalytic tyrosine residue in the GYISYPRTET motif are conserved, PfTopo III possesses a unique charged 85-amino-acid long stretch in domain II (Supplementary Figure S1A). This domain is absent in all eukaryotic Topo III but, bacterial Topo III has a similar charged loop, albeit smaller in length (17 amino acids). The molecular dynamic simulations of PfTopo III with a single-stranded DNA octamer (5′ CGCAACTT 3′) show that the enzyme undergoes a conformational change upon DNA-binding, generating a central cavity, that resembles a ‘protein-mediated DNA gate’ [29]. Charged residues in the enzyme directly participate in hydrogen bonding and stacking interactions with the oligonucleotides, helping to stabilize DNA binding [29].

Yeast two-hybrid analysis shows that PfTopo III associate with both the RecQ helicases, PfBLM (Bloom syndrome protein) and PfWRN (Werner syndrome protein), in the parasite. However, RMI has not been identified in *Plasmodium* [30]. PfTopo III expression is tightly linked to parasite replication [29]. In response to treatment with hydroxy urea (HU), which inhibits the ribonucleotide reductase, parasite growth and replication are arrested with concomitant induction of PfTopo III. Genetic studies indicate that the charged domain is critical for enzyme activity. While a transgenic parasite line with ectopically expressed PfTopo III can rescue HU toxicity, a transgenic parasite line expressing the charged domain deletion mutant cannot. In addition, while full-length PfTopo III can reverse the slow growth phenotype and MMS sensitivity of the *∆topoIII* yeast strain, the charged domain deletion mutant of PfTopo III cannot [29]. It has been proposed that the charged domain is important for effective binding of the enzyme to DNA. A chromatin immunoprecipitation study showed that the charged domain deletion mutant had less of an association with DNA than the full-length enzyme.

PfTopo III exhibits both nuclear and mitochondrial localization [29]. *Plasmodium* mitochondria undergo rolling circle replication and electron microscopy has revealed a complex network of linear concatemers [31]. PfTopo III is primarily recruited to the terminal end of the mtDNA during the late schizont stage. This indicates that it is likely to play a role in the decatenation of mtDNA during segregation. PfTopo III is also reported to interact strongly with PfRad51 [32]. A recent study has established that PfRad51 and PfBLM are both imported into *Plasmodium* mitochondria to repair the mitochondrial genome [32]. The interaction between PfTopo III, PfBLM, and PfRad51 suggest that PfTopo III is a component of the ‘recombinosome’ complex and plays a role in mitochondrial DNA replication and repair.

### Role of topoisomerase IB in *Plasmodium* biology

Multiple sequence alignment shows high variability in length of putative Topo IB sequences within different apicomplexan parasites, where *Theileria annulata*, *Toxoplasma gondii*, and *P. falciparum* Topo IB proteins are much larger in length, while *Babesia bovis* and *Cryptosporidium parvum* orthologs are similar in size to human Topo IB [33]. This heterogeneity is primarily the result of variable lengths in the core and N-terminal domains of distinct apicomplexan parasites. In *P. falciparum*, Topo IB (PF3D7_0510500) encodes for an 839 amino acid protein [34]. Pairwise alignment reveals that PfTopo IB has 42.2% sequence identity with the human ortholog. PfTopo IB has considerably shorter amino-terminal domain than HsTopo IB. The linker region, connecting the amino-terminal and carboxy terminal domains of PfTopo IB is longer than the human counterpart by 16 residues and shares good sequence similarity. Using a hybrid enzyme in which the human Topo IB linker was replaced with the *Plasmodium* Topo IB linker domain showed that the re-ligation rate of human Topo IB can be modulated by altering the length of the linker domain. The hybrid enzyme has faster kinetics of re-ligation and CPT resistance [35]. The C-terminus of PfTopo IB shares high sequence similarity with the human ortholog and possesses a consensus motif, LGTSKINYMDPR, that surrounds the active site tyrosine residue. However, there are three unique stretches of amino acids (I–III) in the core domain of PfTopo IB, consisting of 11 amino acids (I), 29 amino acids (II), and 79 amino acids (III), respectively, that are absent in the human ortholog (Supplementary Figure S1B). These unique stretches are not in the low complexity region and may be important for PfTopo IB activity. Thus, structure-function analysis of PfTopo IB requires further attention. The PfTopo IB promoter is inactive at the ring stage and shows activity in the late trophozoite and schizont stages indicating that it has a specific function in DNA replication [36].

## Type II DNA topoisomerase

### Prelude

Type II DNA topoisomerases are evolutionarily conserved and essential for the survival of every living organism. They are dimeric enzyme and are classified as Type IIA or Type IIB depending on their domain organization. Type IIA enzymes form a homodimer and utilize three protein interphases during DNA decatenation, N-gate, DNA-gate, and C-gate. Type IIB enzymes form a hetero-tetramer (A_2_B_2_) with two subunits: A and B. The B subunit possesses an ATPase domain while the A subunit includes the DNA binding domain and cleavage domain. These enzymes are much simpler in structure compared to that of Type IIA enzymes, utilizing two protein gates to execute their function and lacking a C-gate. Both enzyme families have similar ATPase domains and N-gate structures. The ATPase domain folds into the Bergerat fold and is identical to the GHKL ATPase domain (Gyrase, Hsp90, Histidine kinase, and MutL). The DNA cleavage and re-joining domain, TOPRIM (Topoisomerase-Primase), and DNA binding domain, CAP (Catabolite Activator Protein), also share considerable similarities between the two sub-classes. To change the topology of DNA, the enzyme binds to the first DNA duplex (G-segment) and allows passage of the second DNA duplex (T-segment) into the enzyme cavity [37]. ATP binding then causes dimerization of the amino-terminal domain to close the entry gate (N-gate) along with transient cleavage of the G segment and release of the T segment through the break. The G segment is then ligated. The Type IIA enzyme allows the T segment to be expelled through the third gate, known as the C-gate, while the Type IIB enzyme directly releases the T segment once it passes through the G gate.

Topoisomerase II not only resolves topological perturbations during DNA replication and transcription but also aids in the decatenation of sister chromatids prior to mitosis [13]. This is one of the primary components of the mitotic chromosome scaffold [38] and plays a critical role in establishing and maintaining condensed chromatin during the pro-metaphase stage of mitosis. Depletion of Topo IIα in human cells leads to chromatin entanglement during prometaphase, resulting in chromosome structure deformity and premature exit from mitosis [39]. In contrast, Topo IIβ has no role during the early mitotic phase but is instead involved in the transcription of subsets of genes [40,41]. During neuronal stimulation, Topo IIβ-mediated DNA double-strand breaks (DSB) are generated in the promoters of early-response genes that help to resolve the topological barrier so that RNAPII can move forward [42].

DNA gyrases are present in bacteria, plants, archaea, and apicomplexan parasites. They not only catalyze the easing of positively supercoiled DNA ahead of a replication fork [43] but also have a unique ability to introduce negative supercoils into the relaxed DNA [44]. Bacterial gyrases are also involved in catalysing the decatenation of newly replicated DNA along with topoisomerase IV [45] and play a critical role in chloroplast nucleoid partitioning in plants [46]. This enzyme can cause chromosome condensation and loss of function gyrase mutants show a dramatic reduction in chromosome supercoiling and a lower rate of transcription elongation in the genome [47]. Using a next-generation sequencing approach, gyrase cleavage sequences are shown to be enriched in the transcription termination sites of highly transcribed operons; however, the distribution of gyrases is altered in the presence of the Rifampicin, underscoring the importance of gyrase in regulating transcription [48].

Topoisomerase VI was the sole member of the evolutionary distinct family of type IIB topoisomerase until two additional members were recently identified, Topo VIII and Mini-A [49]. Topo VI was first identified in archaea [50] and later, detected in plants [51–54]. In plants, Topo VI is responsible for the decatenation of DNA during endoreduplication [53]. Mutations in *hyp6*, that encodes for *Arabidopsis thaliana* AtTopo VIB and *rhl2*, that encodes for AtSPO11-3, result in an extreme dwarf phenotype and the mutants display only 8C nuclei, unlike the 32C nuclei seen in the wild-type plant [53]. In addition, in *A. thaliana* and the monocot rice plant, *Oryza sativa*, Topo VIB and Spo11 function as a meiotic snip and the catalytic core complex responsible for initiating the meiotic double-strand break adopt a TopoVI-like structure [55,56]. In most higher eukaryotes, Topo VIB subunits are not found. It is thought that Topo VI evolved before the divergence of eukaryotic and archaeal lineages and Topo VIA was adapted as Spo11, which performs a similar enzymatic function as the Topo VI holoenzyme [57,58]. Spo11 has a specific function in meiosis, initiating meiotic recombination by causing a double-strand break in the chromosome [59–61]. Recently, a Topo VIBL (VIB like) protein was identified in mice (homolog of human C11orf80), that shares 11% sequence identity with the archaeal ortholog [62]. In addition, MmTopo VIBL was found to have a direct role in meiotic double-strand break formation in mice. Homozygous Topo VIBL^−/−^ mice display a dramatic reduction in meiotic double-strand break formation during prophase along with defective oocyte and spermatocyte development, a phenotype similar to that seen in Spo11^−/−^ mice [62].

*Plasmodium* harbours two Type IIA enzymes, Topoisomerase II and gyrase, and one Type IIB subfamily enzyme, Topoisomerase VI.

### Role of topoisomerase II in *Plasmodium* biology

**Figure 2:**
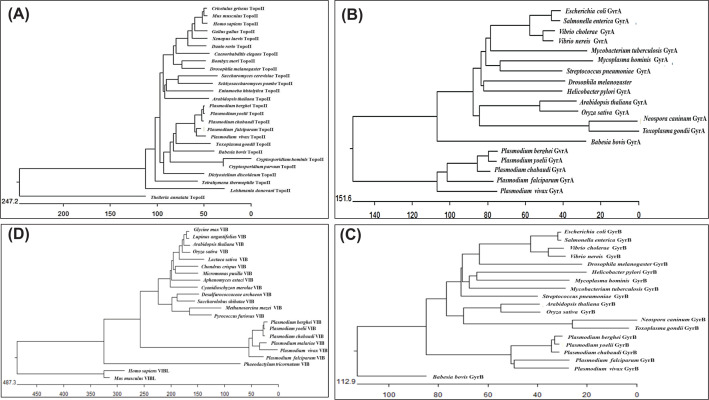
Molecular phylogenetic analysis of Type II DNA topoisomerases (**A**) Phylogenetic tree was constructed using amino acid sequences of topoisomerase II from apicomplexan parasites, and higher eukaryotic organisms (**B**) similarly, it was constructed using amino acid sequences of Gyrase subunit A from apicomplexan parasites and other organisms (**C**) similar to that of B), it was constructed using amino acid sequences of Gyrase subunit B from apicomplexan parasites and other organisms (**D**) similar to the above cases, the tree was constructed using amino acid sequences from TopoVIB in apicomplexan parasites and other organism.

Phylogenetic analysis illustrates that PfTopo II is similar to its homolog in *Plasmodium* species but distinct from that of other apicomplexan parasites, especially *T. annulata* (Figure 2A). PfTopo II encodes a protein with 1,472 amino acids that shares 40% identity with the human Topo IIα. The N-terminal ATPase domain (1-469 amino acids) has a consensus GFGAKLTNIFSKEF motif that is critical for ATP hydrolysis; however, there are two sequentially arranged asparagine rich insertions in the ATPase domain that are unique to *Plasmodium* and absent in human and other apicomplexan parasites (Supplementary Figure S1C). Unlike other replication proteins, which are not expressed during the ring or early trophozoite stages of the malaria parasite, this enzyme is expressed in all intraerythrocytic development stages [63]. Expression of PfTopo II was challenging in the heterologous bacterial system. However it could be purified following codon optimization and using a wheat cell-free protein translation system [64]. Biochemical studies indicate that the central core domain (470−1212 amino acids) is associated with DNA breakage and re-joining activities. Intriguingly, truncated individual domains fail to reconstitute the functional enzyme, indicating that the decatenation activity of PfTopo II requires a covalent association between the two domains. Nevertheless, the C-terminal (1213−1472 amino acids) does not appear to be critical for the catenation/decatenation activity of PfTopo II, and deletion of this domain enhances enzyme stability [64].

### Role of gyrase in *Plasmodium* biology

Phylogenetic analysis suggests that, like other topoisomerases, PfGyrA and PfGyrB are also closely related between *Plasmodium* species but distinct from other apicomplexan parasites and prokaryotic species (Figure 2B,C). In *P. falciparum*, PfGyrA (PF3D7_1223300) and PfGyrB (PF3D7_1239500) encode proteins containing 1,222 and 1,006 amino acids, respectively. PfGyrA and PfGyrB share 30% and 28% sequence identity with their respective homologs in *E. coli*, respectively. The amino acid sequence of PfGyr is significantly different from its bacterial counterparts; however, the functional domains are retained. PfGyrA possesses the DNA cleavage and re-joining domains along with a conserved C-terminal domain responsible for DNA supercoiling. While the Gyr-A box is absent in PfGyrA, it has two unique stretches (containing 49 and 135 amino acids) in low-complexity regions whose function remains obscure. Bioinformatics analysis suggests that the β-propeller domain of PfGyrA contains fewer propellers than its bacterial counterpart [65,66]. Unfortunately, full-length PfGyrA has not been biochemically characterized because it is difficult to express and purify.

PfGyrB has been successfully expressed, purified, and biochemically characterized. This enzyme has conserved ATPase and TOPRIM domains at the amino-terminal and carboxy-terminal ends, respectively. PfGyrB possesses two stretches of low-complexity regions of lengths 124 and 24 amino acids, which are absent in other gyrases. Interestingly, PfGyrB has a strong intrinsic ATPase activity which is stimulated by bacterial GyrA and/or dsDNA. Moreover, PfGyrB in association with the WHD (Winged-Helix Domain) and tower domains of PfGyrA can supercoil the relaxed DNA and efficiently cleave the DNA [67]. In addition, it was demonstrated that Ciprofloxacin stabilizes the hybrid enzyme-bound nicked DNA complex and prevents it from re-joining in a dose-dependent manner. There is a 45 amino acid-long unique insertion within the TOPRIM domain of PfGyrB that is shown to be indispensable for DNA binding, DNA-induced ATPase activity, and DNA cleavage activity of PfGyrase [68]. A genetic complementation assay showed that the PfGyrB∆45 mutant fails to rescue the survival of the temperature-sensitive bacterial gyrase B mutant.

Both PfGyrA and PfGyrB contain a unique 165 and 120 amino acid containing amino terminal extension, respectively, that harbors an apicoplast targeting signal and are localized in the apicoplast [67] but not in the mitochondria [69]. Both subunits are expressed in the late schizont stage of the parasite, although there is only about one enzyme for every two apicoplast genomes [70]. The importance of *Plasmodium* gyrase A was recently established by functional inactivation of PfGyrA in the drug-resistant *P. falciparum* Dd2 strain using a CRISPR-Cas9 gene-editing system [71]. The knockout parasite survived in the presence of isopentenyl pyrophosphate (IPP) and had an intact nucleus and mitochondrion but complete loss of the apicoplast genome. The knockout parasites proliferated normally in the presence of IPP but died within 72 h after IPP removal. Interestingly, Dd2∆GyrA displayed a delayed-death phenotype in response to Ciprofloxacin treatment that could not be rescued by IPP supplementation, indicating that this drug may have a non-apicoplast target in the parasite [71].

### Role of topoisomerase VI in *Plasmodium* biology

*P. falciparum* harbor a functional Topo VIB (Pf3D7_1365600) and Spo11 (PfTopo VIA) (Pf3D7_1217100.1) that encode 561 and 327 amino acid proteins, respectively [72]. However, another putative paralog, Spo11-2 (PF3D7_1027600), exists that encodes for a protein with 336 amino acids. PfTopo VIB shares 31.5% similarity with SsTopo VIB. Phylogenetic analysis shows that during evolution, the Topoisomerase VIB of *P. falciparum* remains closely related to its orthologs in other *Plasmodium* species but distinct from other archaea species and higher eukaryotes (Figure 2D). The accession numbers of Topo II, Gyrase A and B subunits, Topo VIB from various organisms used in our analysis are presented in the Supplementary Data.

Because there is no significant sequence conservation between PfTopo VIB and the Topo VIB−like protein in humans, a dot plot cannot be drawn. In the *Plasmodium* topoisomerase, VIB, the ATP-binding domain, which spans from amino acids 22 to 166, is homologous to the one in the GHKL ATPase protein family that constitutes the Bergerat fold [72]. There is a unique stretch of highly charged amino acids spanning 61−78 within the ATPase domain [73] whose function has not yet been identified. PfTopo VIA harbors the CAP and TOPRIM domains that share 34.8% and 57% sequence similarity with SsTopo VIA, respectively. Both PfTopo VIA and PfTopo VIB are expressed during the schizont stage of parasite development and the PfTopo VIB protein is localized in both the nuclear and organelle fractions, suggesting that it has a possible role in parasite replication. Yeast two-hybrid analysis indicated that the full-length PfTopo VIB strongly interacts with PfTopo VIA. Genetic evidence suggests that both PfTopo VIA and PfTopo VIB possess Type II topoisomerase-like activity because these subunits together can rescue the lethal phenotype of yeast *∆topoII* mutants, while PfTopo VIB alone cannot [72]. Unfortunately, purification of both proteins has been unsuccessful to date. The yeast cell extract that harbours the PfTopo VIB–VIA complex as a sole Type II topoisomerase in *∆topoII* strain was used to evaluate its enzymatic activity. It was observed that the PfTopo VIB–VIA complex can relax supercoiled plasmid DNA and decatenate kDNA in an ATP and magnesium-dependent manner [72].

## Inhibitors against *Plasmodium* topoisomerases

A complete list of inhibitors against *Plasmodium* topoisomerases is presented in Table 4. There are two types of drugs known to target Topo IB: the class I inhibitors stabilize the DNA–enzyme complex post DNA-cleavage, whereas class II inhibitors obstruct the catalytic activity of the enzyme. One of the most remarkable class I inhibitors is the pentacyclic alkaloid, Camptothecin (CPT). CPT inhibits the plasmid relaxation activity of PfTopo IB in a dose-dependent manner [36] and traps the protein–DNA complex, subsequently inhibiting nucleic acid biosynthesis in the parasite and causing cell death [74]. A modified isoflavonoid, N-tosyl-azapterocarpan (LQB223), is predicted to bind to the CPT-binding pocket of PfTopo IB, showing a high selectivity index and probable anti-parasitic activity *in vivo* [75]. In another study, a synthetic octapeptide, WRWYCRCK, was designed that is predicted to bind to the interface between non-covalent DNA and PfTopo IB and serve as a representative class II inhibitor. The docking study revealed that the peptide was stabilized by various interactions between the enzyme and DNA, and was predicted to prevent the catalytic tyrosine from forming a nick in the DNA that corelates with the inhibition of DNA cleavage activity in a dose-dependent manner [76]. However, because the peptide was not predicted to bind to the covalent enzyme-bound-DNA complex, the ligation step remained unaffected by the peptide.

While the domain organizations and enzymatic activities of Type IIA and Type IIB topoisomerase are similar, there are distinct differences in their mechanisms of action. Thus, most inhibitors that target Type IIA topoisomerases are inactive against Topo IIB. The unknotting activities of purified Topo II from *P. falciparum* are susceptible to both prokaryotic and eukaryotic type II topoisomerase inhibitors [64]. Etoposide treatment causes double-stranded DNA breaks in a purified PfTopo II−mediated plasmid relaxation assay and in the *P. falciparum* genome [77]; however, it fails to exhibit significant selective inhibition of purified PfTopo II over human Topo II. Ciprofloxacin treatment, in contrast, displayed more than 50-fold selective inhibition of PfTopo II over human Topo II. Another class of bacterial gyrase inhibitor, piperidinyl-alkyl-quinoline (GSK299423), showed 15-fold inherent selectivity for PfTopo II over its human counterpart and, unlike etoposide, resulted in asymmetric single-stranded DNA breaks at the DNA binding site [64]. Ciprofloxacin and GSK299423 have similar selectivity for the 3D7 parasite culture as human macrophages. In one study, chloroquine and pyrimethamine resistant *Plasmodium* K1 strain extracts were used to screen various 9-anilinoacridine analogues for their ability to inhibit the decatenation activity of type II topoisomerases. One of the derivatives, 3,6-diamino-1'-amino-9-anilinoacridine, was identified as the most effective, with a IC_50_ of 25 nM in parasite culture and more than 600-fold selectivity toward parasite culture as compared to human leukaemia cells [78]. However, the mechanism of action for this inhibition was not demonstrated.

Inhibition of *Plasmodium* gyrase by Ciprofloxacin causes cleavage of the apicoplast genome [79]. Although Ciprofloxacin does not inhibit apicoplast genome segregation in the progeny, antibiotic-treated merozoites possess morphologically abnormal apicoplasts and exhibit delayed death [80]. Several quinolones derivatives were tested for their efficacy including whether they could promote the formation of linearised apicoplast DNA in an *in vitro Plasmodium* culture and Clinafloxacin was identified as the most potent inhibitor [70]. Novobiocin was also shown to inhibit the ATPase activity of purified PfGyrB in a dose-dependent manner, which correlated with a decrease in parasitaemia particularly in the second cycle of parasite growth. This drug blocked the transition from the trophozoite to schizont stages and reduced the apicoplast copy number at the schizont stage, underscoring the importance of gyrase to parasite survival [69]. Recently, a chemical library that showed specific binding to PfGyrB but not to EcGyrB, was used to test its efficacy against the ATPase activity, supercoiling, and cleavage activity of the hybrid enzyme, PfGyrB-EcGyrA. This experiment helped in the identification of a novel inhibitor, purpurogallin (PPG) [81]. PPG inhibits the DNA binding activity of both PfGyrB and EcGyrA-PfGyrB as well as the DNA supercoiling activity and ATP hydrolysis of the hybrid enzyme. However, the efficacy of PPG toward the parasite blood stage was low with an IC_50_ of only100 μM.

Radicicol and Etoposide inhibit the decatenation activity of PfTopo VI in a dose-dependent manner, but Novobiocin has no effect on the enzyme [72]. Radicicol was previously shown to inhibit archaeal Topo VI−mediated decatenation of kDNA and relaxation of supercoiled plasmid DNA [82]. X-ray crystallography was used to determine the structural basis for Topo VI inhibition by Radicicol. It was found that Radicicol competes with ATP for binding to the ATPase pocket (Bergerat fold) of Topo VIB, effectively blocking nucleotide-mediated dimerization of the Topo VIB subunits [83]. Using the SsTopo VIB structure as a template, an *in silico* model of PfTopo VIB was built and Radicicol was found to dock in the ATP binding pocket of PfTopo VIB, similar to SsTopo VIB [73]. When tested in a *P. falciparum* 3D7 culture, Radicicol inhibited parasite growth with an IC_50_ of 8 μM [84]. However, at sublethal doses, Radicicol reversibly arrested the parasites in the schizont stage and prevented its transition to the ring stage. While Radicicol did not change the ploidy of the treated parasite, it substantially reduced its mitochondrial genome content even at sublethal doses [84]. The above studies suggest that the target protein of Radicicol is redundant for nuclear replication but essential for mitochondrial replication.

## Unanswered questions

**Figure 3:**
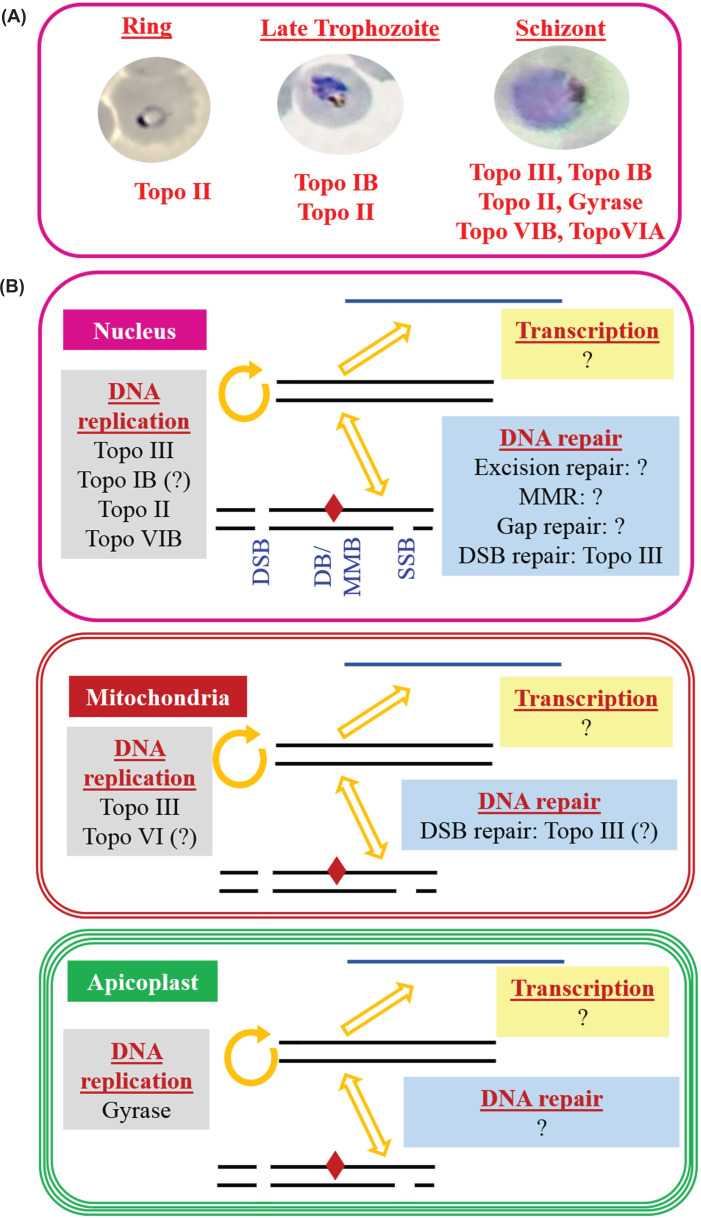
Expression and activities of *Plasmodium* topoisomerases at the asexual stage of the parasite (**A**) Stage specific expression of various topoisomerases is presented. (**B**) The identified functions of *Plasmodium* topoisomerases at the nucleus, mitochondria and apicoplast are presented. The unexplored role of the topoisomerases at various processes of DNA metabolism are schematically presented as (?); DB, damaged base; DSB, double strand break; MMB, mismatched base; MMR, mismatch repair; SSB, single strand break.

*Plasmodium* topoisomerase sequences show significant variation from their human homologs (Supplementary Figure S1). Hence, additional work is needed to gain detailed insight into the structural properties of these enzymes so that a rational approach for drug design can be adopted. In addition, the biological function of each topoisomerase in various stages of DNA metabolism remains largely unknown (Figure 3). The absence of gyrase and topoisomerase VI in humans could make these enzymes good targets for treating parasite infection. PfGyrB has been extensively characterized; however, the structure–function analysis of PfGyrA and PfTopo VIB remain pending. Such studies could aid the development of novel inhibitors of *P. falciparum*. The structure–function analysis of PfTopo III should help to elucidate the function of the charged domain of *Plasmodium* Topo III. Because this domain is unique to the sequence of PfTopo III and essential for its *in vivo* enzyme activity, it qualifies as a novel target for malaria. Because RMI is absent from the *Plasmodium* genome, it remains unknown how RecQ helicases alone can stimulate the activity of PfTopo III. Topo IIIα is critical to the survival of fission yeast [85] and Topo IIIα deletions lead to embryogenic lethality in mice [86], but whether this enzyme is essential to the *Plasmodium* life cycle remains to be addressed. Topo IIIα deficiency increases the antigenic switching frequency in Trypanosoma at the variant surface glycoprotein (VSG) locus in a Rad51-dependent manner [87]. However, whether PfTopo III also regulates var gene switching in *P. falciparum* needs to be explored in order to control its virulence.

Topo IB and Topo II undergo several post-translational modifications (PTMs), which regulate their activity. Human Topo IB is physically associated with O-linked β-N-acetylglucosamine transferase and forms O-GlcNAc-TopoIB *in-vivo* [88]. The higher level of O-GlcNAc-TopoIB is linked to an increase in its DNA relaxation activity. Human Topo IIα undergoes several PTMs including phosphorylation, acetylation, ubiquitination and SUMOylation, which help to regulate its function [89,90]. Human Topo IB and Topo II undergo differential phosphorylation in a cell cycle-dependent manner [91,92]. The dephosphorylation of the mammalian Topo IB completely inhibits its DNA relaxation activity and increases its sensitivity to CPT [93]. PfTopo IB and PfTopo II possess several putative phosphorylation sites and it is speculated that phosphoprotein may regulate the *in vivo* function of Topo IB. However, the extent of PTMs of these enzymes and how these changes influence their structure and function remains unknown. Genome-wide Topo IB cleavage sites were recently identified and compared between CPT treated and untreated human colon cancer cell lines (HCT116) [28] and Topo IB was found to predominantly engage the promoters. CPT was also shown to synergize with the bromodomain inhibitors. No such studies have been performed, to determine whether PfTopo IB plays a similar role in regulating *Plasmodium* transcription.

The *in vivo* function of Topo IIα is modulated by a few novel interacting proteins. Human Topo IIα remains associated with both Hsp90α and Hsp90β isoforms of heat shock protein-90, HOP (Sti1), Hsc70, and Grp94 [94]. Interestingly, Hsp90 inhibitor (geldanamycin) in combination with topoisomerase poison (etoposide and mitoxantrone) had a synergistic effect on the proliferation of different tumour cell types both *in vivo* and *in vitro*. Whether PfTopo II also interacts with *Plasmodium* chaperones remains obscure. The relationship between PfHsp90 and PfTopo II, may reveal new ways to target this topoisomerase in malaria parasites. In a similar line, a recent study reveals that simultaneous targeting of Hsp90 and recombinase results in extreme synergy growth defect in both drug-sensitive (3D7) and drug-resistant (Dd2) malaria parasites [95].

Radicicol-induced inhibition of the parasitic mitochondrial genome [84] indicated that PfTopo VI might play a role during mitochondrial DNA decatenation, a process that is necessary for endoreduplication. However, more research is required to further explore this. It is also important to determine which Spo11 paralog actually forms a holoenzyme with PfTopo VIB in the asexual stage of the parasite. It may be technically challenging to create a conditional knockout of PfTopo VI given that it may be essential for parasite survival. Thus, highly specific chemical inhibitors of PfTopo VIB should be employed to characterize its precise role in *Plasmodium* biology. Radicicol holds promise as an inhibitor of PfTopo VIB but has off-target activity within the parasite. In a recent study, the binding affinities of 98 Radicicol derivatives with the *in silico* models of PfTopo VIB and PfHsp90 were predicted. Some derivatives were predicted to bind strongly with PfTopo VIB but not with PfHsp90 [73]. These approaches can be used to design specific PfTopo VIB inhibitors which improve the understanding of the PfTopo VIB's role in malaria parasite biology.

## Conclusions

Characterization of *Plasmodium* topoisomerases is still in its infancy. Although, the biological functions of some topoisomerases have been studied in the asexual stages of the parasite (Figure 3), there are no reports to date about their function in the sexual and liver stages. Topo VI, Topo III, and Topo II have bona fide roles in meiosis since they catalyze the initiation of double-strand breaks, resolve Holliday junctions during meiotic recombination, and segregate recombined chromosomes, respectively. It will be particularly important to identify whether they perform similar functions during the sexual stage of *Plasmodium* and whether it would be worthwhile to target these enzymes as part of a strategy to block malaria transmission. To study the function of topoisomerases during meiosis, conditional knockout strains will need to be generated. *Plasmodium berghei* may be an excellent model because the sexual stage-specific conditional knockout approach has been well developed in this rodent parasite model.

Several advancements have been made to validate topoisomerase as an effective drug target against many bacterial pathogens. However, a more in-depth study is required for the development of novel therapeutic agents that specifically target *Plasmodium* topoisomerases. Purifying these proteins, however, has remained unsuccessful. Distinct approaches like codon harmonization or specific expression systems should be utilized to express and purify *Plasmodium* topoisomerases in order to perform biochemical and functional analysis of these proteins.

## Supplementary Material

Supplementary Figure S1

Supplementary Data

## Abbreviations

CPT: Camptothecin
HU: hydroxy urea
IPP: isopentenyl pyrophosphate
PPG: purpurogallin
PTM: post-translational modification
RNAPII: RNA polymerase II
TSS: transcription start site
TTS: transcription termination site

## References

[1] Organization, W.H.. World malaria report 2021., 2021

[2] Venugopal K. et al. (2020) Plasmodium asexual growth and sexual development in the haematopoietic niche of the host. Nat. Rev. Microbiol. 18, 177–189 10.1038/s41579-019-0306-2 31919479 PMC7223625

[3] Diffley J.F. (2004) Regulation of early events in chromosome replication. Curr. Biol. 14, R778–R786 10.1016/j.cub.2004.09.019 15380092

[4] Reilly Ayala H.B. et al. (2007) Quantitative dissection of clone-specific growth rates in cultured malaria parasites. Int. J. Parasitol. 37, 1599–1607 10.1016/j.ijpara.2007.05.003 17585919 PMC2268714

[5] Murray L. et al. (2017) Multiplication rate variation in the human malaria parasite Plasmodium falciparum. Sci. Rep. 7, 1–8 10.1038/s41598-017-06295-9 28743888 PMC5527095

[6] Matthews H., Duffy C.W. and Merrick C.J. (2018) Checks and balances? DNA replication and the cell cycle in Plasmodium Parasites Vectors 11, 1–13 10.1186/s13071-018-2800-1 29587837 PMC5872521

[7] Wang J.C. (2002) Cellular roles of DNA topoisomerases: a molecular perspective. Nat. Rev. Mol. Cell Biol. 3, 430–440 10.1038/nrm831 12042765

[8] Postow L. et al. (2001) Topological challenges to DNA replication: conformations at the fork. Proc. Natl. Acad. Sci. 98, 8219–8226 10.1073/pnas.111006998 11459956 PMC37424

[9] Racko D. et al. (2018) Chromatin loop extrusion and chromatin unknotting. Polymers 10, 1126 10.3390/polym10101126 PMC6403842 30961051

[10] García M.T. et al. (2011) New alkaloid antibiotics that target the DNA topoisomerase I of Streptococcus pneumoniae. J. Biol. Chem. 286, 6402–6413 10.1074/jbc.M110.148148 21169356 PMC3057782

[11] Godbole A.A. et al. (2015) Targeting Mycobacterium tuberculosis topoisomerase I by small-molecule inhibitors. Antimicrob. Agents Chemother. 59, 1549–1557 10.1128/AAC.04516-14 25534741 PMC4325804

[12] Kongsoi S. et al. (2015) Characterization of Salmonella Typhimurium DNA gyrase as a target of quinolones. Drug Test. Anal. 7, 714–720 10.1002/dta.1744 25381884

[13] Nitiss J.L. (2009) Targeting DNA topoisomerase II in cancer chemotherapy. Nat. Rev. Cancer 9, 338–350 10.1038/nrc2607 19377506 PMC2748742

[14] Chowdhury S.R. and Majumder H.K. (2019) DNA Topoisomerases in unicellular pathogens: structure, function, and druggability. Trends Biochem. Sci. 44, 415–432 10.1016/j.tibs.2018.12.001 30609953

[15] McKie S.J., Neuman K.C. and Maxwell A. (2021) DNA topoisomerases: Advances in understanding of cellular roles and multi‐protein complexes via structure‐function analysis. Bioessays 43, 2000286 10.1002/bies.202000286 PMC7614492 33480441

[16] Slesarev A.I. et al. (1993) DNA topoisomerase V is a relative of eukaryotic topoisomerase I from a hyperthermophilic prokaryote. Nature 364, 735–737 10.1038/364735a0 8395022

[17] Taneja B. et al. (2006) Structure of the N‐terminal fragment of topoisomerase V reveals a new family of topoisomerases. EMBO J. 25, 398–408 10.1038/sj.emboj.7600922 16395333 PMC1383508

[18] Kim R.A. and Wang J.C. (1989) Function of DNA topoisomerases as replication swivels in Saccharomyces cerevisiae. J. Mol. Biol. 208, 257–267 10.1016/0022-2836(89)90387-2 2549254

[19] Heyer W.-D., Ehmsen K.T. and Solinger J.A. (2003) Holliday junctions in the eukaryotic nucleus: resolution in sight? Trends Biochem. Sci. 28, 548–557 10.1016/j.tibs.2003.08.011 14559184

[20] Tsai H.-J. et al. (2006) Involvement of topoisomerase III in telomere-telomere recombination. J. Biol. Chem. 281, 13717–13723 10.1074/jbc.M600649200 16546998

[21] Hu P. et al. (2001) Evidence for BLM and topoisomerase IIIα interaction in genomic stability. Hum. Mol. Genet. 10, 1287–1298 10.1093/hmg/10.12.1287 11406610

[22] Wu L. et al. (2000) The Bloom's syndrome gene product interacts with topoisomerase III. J. Biol. Chem. 275, 9636–9644 10.1074/jbc.275.13.9636 10734115

[23] Nicholls T.J. et al. (2018) Topoisomerase 3α is required for decatenation and segregation of human mtDNA. Mol. Cell 69, 9.e6–23. e6 10.1016/j.molcel.2017.11.033 29290614 PMC5935120

[24] Ahmad M. et al. (2017) Topoisomerase 3β is the major topoisomerase for mRNAs and linked to neurodevelopment and mental dysfunction. Nucleic Acids Res. 45, 2704–2713 28039324 10.1093/nar/gkw1293 PMC5389537

[25] Joo Y. et al. (2020) Topoisomerase 3β knockout mice show transcriptional and behavioural impairments associated with neurogenesis and synaptic plasticity. Nat. Commun. 11, 1–18 10.1038/s41467-020-16884-4 32561719 PMC7305123

[26] Jain T., Roper B.J. and Grove A. (2009) A functional type I topoisomerase from Pseudomonas aeruginosa. BMC Mol. Biol. 10, 1–13 10.1186/1471-2199-10-23 19317906 PMC2666729

[27] Champoux J.J. and Dulbecco R. (1972) An activity from mammalian cells that untwists superhelical DNA—a possible swivel for DNA replication. Proc. Natl. Acad. Sci. 69, 143–146 10.1073/pnas.69.1.143 4333036 PMC427563

[28] Baranello L. et al. (2016) RNA polymerase II regulates topoisomerase 1 activity to favor efficient transcription. Cell 165, 357–371 10.1016/j.cell.2016.02.036 27058666 PMC4826470

[29] Bansod S. et al. (2020) Elucidation of an essential function of the unique charged domain of Plasmodium topoisomerase III. Biochem. J. 477, 4745–4767 10.1042/BCJ20200318 33241842

[30] Lee A.H., Symington L.S. and Fidock D.A. (2014) DNA repair mechanisms and their biological roles in the malaria parasite Plasmodium falciparum. Microbiol. Mol. Biol. Rev. 78, 469–486 10.1128/MMBR.00059-13 25184562 PMC4187680

[31] Preiser P. et al. (1996) Recombination associated with replication of malarial mitochondrial DNA. EMBO J. 15, 684–693 10.1002/j.1460-2075.1996.tb00401.x 8599952 PMC449987

[32] Jha P. et al. (2021) Bloom helicase along with recombinase Rad51 repairs the mitochondrial genome of the malaria parasite. Msphere 6, e00718–e00721 10.1128/mSphere.00718-21 PMC8565512 34730376

[33] García-Estrada C. et al. (2010) DNA topoisomerases in apicomplexan parasites: promising targets for drug discovery. Proc. Royal Soc. B: Biol. Sci. 277, 1777–1787 10.1098/rspb.2009.2176 PMC2871873 20200034

[34] Tosh K. and Kilbey B. (1995) The gene encoding topoisomerase I from the human malaria parasite Plasmodium falciparum. Gene 163, 151–154 10.1016/0378-1119(95)00376-H 7557466

[35] Arnò B. et al. (2013) Replacement of the human topoisomerase linker domain with the plasmodial counterpart renders the enzyme camptothecin resistant. PLoS ONE 8, e68404 10.1371/journal.pone.0068404 23844196 PMC3699648

[36] Tosh K. et al. (1999) Plasmodium falciparum: stage-related expression of topoisomerase I. Exp. Parasitol. 91, 126–132 10.1006/expr.1998.4362 9990340

[37] Lee I., Dong K.C. and Berger J.M. (2013) The role of DNA bending in type IIA topoisomerase function. Nucleic Acids Res. 41, 5444–5456 10.1093/nar/gkt238 23580548 PMC3664819

[38] Earnshaw W.C. et al. (1985) Topoisomerase II is a structural component of mitotic chromosome scaffolds. J. Cell Biol. 100, 1706–1715 10.1083/jcb.100.5.1706 2985625 PMC2113886

[39] Nielsen C.F. et al. (2020) Topoisomerase IIα is essential for maintenance of mitotic chromosome structure. Proc. Natl. Acad. Sci. 117, 12131–12142 10.1073/pnas.2001760117 32414923 PMC7275761

[40] Trotter K.W., King H.A. and Archer T.K. (2015) Glucocorticoid receptor transcriptional activation via the BRG1-dependent recruitment of TOP2β and Ku70/86. Mol. Cell. Biol. 35, 2799–2817 10.1128/MCB.00230-15 26055322 PMC4508321

[41] Tsutsui K. et al. (2001) Involvement of DNA topoisomerase IIβ in neuronal differentiation. J. Biol. Chem. 276, 5769–5778 10.1074/jbc.M008517200 11106659

[42] Madabhushi R. et al. (2015) Activity-induced DNA breaks govern the expression of neuronal early-response genes. Cell 161, 1592–1605 10.1016/j.cell.2015.05.032 26052046 PMC4886855

[43] Tretter E.M. and Berger J.M. (2012) Mechanisms for defining supercoiling set point of DNA gyrase orthologs: I. A nonconserved acidic c-terminal tail modulates escherichia coli gyrase activity*♦. J. Biol. Chem. 287, 18636–18644 10.1074/jbc.M112.345678 22457353 PMC3365713

[44] Gellert M. et al. (1976) DNA gyrase: an enzyme that introduces superhelical turns into DNA. Proc. Natl. Acad. Sci. 73, 3872–3876 10.1073/pnas.73.11.3872 186775 PMC431247

[45] Zechiedrich E.L. and Cozzarelli N.R. (1995) Roles of topoisomerase IV and DNA gyrase in DNA unlinking during replication in Escherichia coli. Genes Develop. 9, 2859–2869 10.1101/gad.9.22.2859 7590259

[46] Cho H.S. et al. (2004) DNA gyrase is involved in chloroplast nucleoid partitioning. Plant Cell 16, 2665–2682 10.1105/tpc.104.024281 15367714 PMC520963

[47] Rovinskiy N. et al. (2012) Rates of gyrase supercoiling and transcription elongation control supercoil density in a bacterial chromosome. PLoS Genet. 8, e1002845 10.1371/journal.pgen.1002845 22916023 PMC3420936

[48] Sutormin D. et al. (2019) Single-nucleotide-resolution mapping of DNA gyrase cleavage sites across the Escherichia coli genome. Nucleic Acids Res. 47, 1373–1388 10.1093/nar/gky1222 30517674 PMC6379681

[49] Takahashi T.S. et al. (2020) Expanding the type IIB DNA topoisomerase family: identification of new topoisomerase and topoisomerase-like proteins in mobile genetic elements. NAR Genomics Bioinform. 2, lqz021 10.1093/nargab/lqz021 PMC7671362 33575570

[50] Bergerat A., Gadelle D. and Forterre P. (1994) Purification of a DNA topoisomerase II from the hyperthermophilic archaeon Sulfolobus shibatae. A thermostable enzyme with both bacterial and eucaryal features. J. Biol. Chem. 269, 27663–27669 10.1016/S0021-9258(18)47037-8 7961685

[51] Hartung F. and Puchta H. (2001) Molecular characterization of homologues of both subunits A (SPO11) and B of the archaebacterial topoisomerase 6 in plants. Gene 271, 81–86 10.1016/S0378-1119(01)00496-6 11410368

[52] Hartung F. et al. (2002) An archaebacterial topoisomerase homolog not present in other eukaryotes is indispensable for cell proliferation of plants. Curr. Biol. 12, 1787–1791 10.1016/S0960-9822(02)01218-6 12401176

[53] Sugimoto-Shirasu K. et al. (2002) DNA topoisomerase VI is essential for endoreduplication in Arabidopsis. Curr. Biol. 12, 1782–1786 10.1016/S0960-9822(02)01198-3 12401175

[54] Yin Y. et al. (2002) A crucial role for the putative Arabidopsis topoisomerase VI in plant growth and development. Proc. Natl. Acad. Sci. 99, 10191–10196 10.1073/pnas.152337599 12119417 PMC126646

[55] Vrielynck N. et al. (2016) A DNA topoisomerase VI–like complex initiates meiotic recombination. Science 351, 939–943 10.1126/science.aad5196 26917763

[56] Fu M. et al. (2016) The DNA Topoisomerase VI-B Subunit OsMTOPVIB Is Essential for Meiotic Recombination Initiation in Rice. Mol. Plant 9, 1539–1541 10.1016/j.molp.2016.07.006 27477684

[57] Gadelle D. et al. (2003) Phylogenomics of type II DNA topoisomerases. Bioessays 25, 232–242 10.1002/bies.10245 12596227

[58] Corbett K.D. and Berger J.M. (2004) Structure, molecular mechanisms, and evolutionary relationships in DNA topoisomerases. Annu. Rev. Biophys. Biomol. Struct. 33, 95–118 10.1146/annurev.biophys.33.110502.140357 15139806

[59] Keeney S., Giroux C.N. and Kleckner N. (1997) Meiosis-specific DNA double-strand breaks are catalyzed by Spo11, a member of a widely conserved protein family. Cell 88, 375–384 10.1016/S0092-8674(00)81876-0 9039264

[60] Celerin M. et al. (2000) Multiple roles of Spo11 in meiotic chromosome behavior. EMBO J. 19, 2739–2750 10.1093/emboj/19.11.2739 10835371 PMC212740

[61] Kee K. and Keeney S. (2002) Functional interactions between SPO11 and REC102 during initiation of meiotic recombination in Saccharomyces cerevisiae. Genetics 160, 111–122 10.1093/genetics/160.1.111 11805049 PMC1461935

[62] Robert T. et al. (2016) The TopoVIB-Like protein family is required for meiotic DNA double-strand break formation. Science 351, 943–949 10.1126/science.aad5309 26917764

[63] Cheesman S. et al. (1994) The gene encoding topoisomerase II from Plasmodium falciparum. Nucleic Acids Res. 22, 2547–2551 10.1093/nar/22.13.2547 8041616 PMC308208

[64] Mudeppa D.G. et al. (2015) Topoisomerase II from human malaria parasites: expression, purification, and selective inhibition. J. Biol. Chem. 290, 20313–20324 10.1074/jbc.M115.639039 26055707 PMC4536438

[65] Nagano S. et al. (2014) Unique features of apicoplast DNA gyrases from Toxoplasma gondii and Plasmodium falciparum. BMC Bioinform. 15, 1–15 10.1186/s12859-014-0416-9 25523502 PMC4297366

[66] Nagano S. et al. (2015) Investigating the roles of the C-terminal domain of Plasmodium falciparum GyrA. PLoS ONE 10, e0142313 10.1371/journal.pone.0142313 26566222 PMC4643928

[67] Dar M.A. et al. (2007) Molecular cloning of apicoplast-targeted Plasmodium falciparum DNA gyrase genes: unique intrinsic ATPase activity and ATP-independent dimerization of PfGyrB subunit. Eukaryot. Cell. 6, 398–412 10.1128/EC.00357-06 17220464 PMC1828931

[68] Dar A. et al. (2009) A unique 45-amino-acid region in the toprim domain of Plasmodium falciparum gyrase B is essential for its activity. Eukaryot. Cell. 8, 1759–1769 10.1128/EC.00149-09 19700639 PMC2772398

[69] Ram E.R. et al. (2007) Nuclear gyrB encodes a functional subunit of the Plasmodium falciparum gyrase that is involved in apicoplast DNA replication. Mol. Biochem. Parasitol. 154, 30–39 10.1016/j.molbiopara.2007.04.001 17499371

[70] Girdwood S.C.T., Nenortas E. and Shapiro T.A. (2015) Targeting the gyrase of Plasmodium falciparum with topoisomerase poisons. Biochem. Pharmacol. 95, 227–237 10.1016/j.bcp.2015.03.018 25881748 PMC4449814

[71] Tan S. et al. (2021) Properties of Plasmodium falciparum with a deleted apicoplast DNA gyrase. Antimicrob. Agents Chemother. AAC.00586–21 10.1128/AAC.00586-21 PMC8370227 34152814

[72] Chalapareddy S. et al. (2016) Radicicol-mediated inhibition of topoisomerase VIB-VIA activity of the human malaria parasite Plasmodium falciparum. Msphere 1, e00025–15 10.1128/mSphere.00025-15 27303712 PMC4863635

[73] Bansod S. et al. (2021) Molecular docking and molecular dynamics simulation identify a novel Radicicol derivative that predicts exclusive binding to Plasmodium falciparum Topoisomerase VIB. J. Biomol. Struct. Dyn. 1–13 10.1080/07391102.2021.1891970 33650468

[74] Bodley A.L., Cumming J.N. and Shapiro T.A. (1998) Effects of camptothecin, a topoisomerase I inhibitor, on Plasmodium falciparum. Biochem. Pharmacol. 55, 709–711 10.1016/S0006-2952(97)00556-X 9515582

[75] Cortopassi W.A. et al. (2014) Theoretical and experimental studies of new modified isoflavonoids as potential inhibitors of topoisomerase I from Plasmodium falciparum. PloS ONE 9, e91191 10.1371/journal.pone.0091191 24651068 PMC3961230

[76] Roy A. et al. (2011) Peptide inhibition of topoisomerase IB from Plasmodium falciparum. Mol. Biol. Int. 2011, 854626 10.4061/2011/854626 PMC3200115 22091414

[77] Kelly J.M., McRobert L. and Baker D.A. (2006) Evidence on the chromosomal location of centromeric DNA in Plasmodium falciparum from etoposide-mediated topoisomerase-II cleavage. Proc. Natl. Acad. Sci. 103, 6706–6711 10.1073/pnas.0510363103 16617116 PMC1458945

[78] Chavalitshewinkoon P. et al. (1993) Structure-activity relationships and modes of action of 9-anilinoacridines against chloroquine-resistant Plasmodium falciparum in vitro. Antimicrob. Agents Chemother. 37, 403–406 10.1128/AAC.37.3.403 8384810 PMC187684

[79] Weissig V., Vetro-Widenhouse T.S. and Rowe T.C. (1997) Topoisomerase II inhibitors induce cleavage of nuclear and 35-kb plastid DNAs in the malarial parasite Plasmodium falciparum. DNA Cell Biol. 16, 1483–1492 10.1089/dna.1997.16.1483 9428797

[80] Dahl E.L. and Rosenthal P.J. (2007) Multiple antibiotics exert delayed effects against the Plasmodium falciparum apicoplast. Antimicrob. Agents Chemother. 51, 3485–3490 10.1128/AAC.00527-07 17698630 PMC2043295

[81] Pakosz Z. et al. (2021) Inhibitory compounds targeting Plasmodium falciparum gyrase B. Antimicrob. Agents Chemother. 65, e00267–21 10.1128/AAC.00267-21 PMC8448092 34339271

[82] Gadelle D. et al. (2005) Inhibition of archaeal growth and DNA topoisomerase VI activities by the Hsp90 inhibitor radicicol. Nucleic Acids Res. 33, 2310–2317 10.1093/nar/gki526 15849317 PMC1084324

[83] Corbett K.D. and Berger J.M. (2006) Structural basis for topoisomerase VI inhibition by the anti-Hsp90 drug radicicol. Nucleic Acids Res. 34, 4269–4277 10.1093/nar/gkl567 16920739 PMC1616964

[84] Chalapareddy S. et al. (2014) Radicicol confers mid-schizont arrest by inhibiting mitochondrial replication in Plasmodium falciparum. Antimicrob. Agents Chemother. 58, 4341–4352 10.1128/AAC.02519-13 24841259 PMC4136038

[85] Maftahi M. et al. (1999) The top 3+ gene is essential in Schizosaccharomyces pombe and the lethality associated with its loss is caused by Rad12 helicase activity. Nucleic Acids Res. 27, 4715–4724 10.1093/nar/27.24.4715 10572171 PMC148771

[86] Li W. and Wang J.C. (1998) Mammalian DNA topoisomerase IIIα is essential in early embryogenesis. Proc. Natl. Acad. Sci. 95, 1010–1013 10.1073/pnas.95.3.1010 9448276 PMC18654

[87] Kim H.-S. and Cross G.A. (2010) TOPO3α influences antigenic variation by monitoring expression-site-associated VSG switching in Trypanosoma brucei. PLoS Pathog. 6, e1000992 10.1371/journal.ppat.1000992 20628569 PMC2900300

[88] Noach N. et al. (2007) Modification of topoisomerase I activity by glucose and by O-GlcNAcylation of the enzyme protein. Glycobiology 17, 1357–1364 10.1093/glycob/cwm105 17932134

[89] Bedez C. et al. (2018) Post-translational modifications in DNA topoisomerase 2α highlight the role of a eukaryote-specific residue in the ATPase domain. Sci. Rep. 8, 1–12 29915179 10.1038/s41598-018-27606-8 PMC6006247

[90] Lotz C. and Lamour V. (2020) The interplay between DNA topoisomerase 2α post-translational modifications and drug resistance. Cancer Drug Resist. 149–160 10.20517/cdr.2019.114 35582608 PMC9090595

[91] D'Arpa P. and Liu L.F. (1995) Cell cycle-specific and transcription-related phosphorylation of mammalian topoisomerase I. Exp. Cell Res. 217, 125–131 10.1006/excr.1995.1071 7867711

[92] Wells N.J. et al. (1995) Cell cycle phase-specific phosphorylation of human topoisomerase IIα: evidence of a role for protein kinase C. J. Biol. Chem. 270, 28357–28363 10.1074/jbc.270.47.28357 7499337

[93] Pommier Y. et al. (1990) Phosphorylation of mammalian DNA topoisomerase I and activation by protein kinase C. J. Biol. Chem. 265, 9418–9422 10.1016/S0021-9258(19)38865-9 2160979

[94] Barker C.R. et al. (2006) The topoisomerase II–Hsp90 complex: a new chemotherapeutic target? Int. J. Cancer 118, 2685–2693 10.1002/ijc.21717 16385570

[95] Tabassum W. et al. (2021) Elevated DNA damage sensitivity in malaria parasite induced by the synergistic action between PfHsp90 inhibitor and PfRad51 inhibitor. Antimicrob. Agents Chemother. AAC.00457–21 10.1128/AAC.00457-21 PMC8370194 34097485

