# Supplementary material for: A tale of topoisomerases and the knotty genetic material in the backdrop of Plasmodium biology
Source: Biosci Rep. 2022 Jun 14;42(6):BSR20212847. doi: 10.1042/BSR20212847 (PMC9261774; doi:10.1042/BSR20212847)
Supplement: Supplementary Figure S1 [file BSR-2021-2847C_supp.pdf]

Supplementary Material

Accession No. of Topo III from various organisms are presented below:

XP_001350185.1 (*P. falciparum*), XP_001614250.1 (*P. vivax*), CEL68917.1 (*N. caninum*), XP_955385.1 (*T. annulata*), XP_019414796.1 (*L. angustifolius*), XP_034423756.1 (*P. berghei* ANKA), Q13472.1 (*H. sapiens*), NP_033436.1 (*M. musculus*), NP_523602.2 (*D. melanogaster*), NP_499558.1 (*C. elegans*), NP_201197.1 (*A. thaliana*), C7J0A2.1 (*O. sativa*), NP_013335.1 (*S. cerevisiae*), NP_596761.1 (*S. pombe*), XP_643754.2 (*D. discoideum*), NP_416277.1 (*E. coli*), XP_728858.2 (*P. yoelii*), XP_016654744.1 (*P. chabaudi chabaudi*), XP_001388262.1 (*C. parvum*), WP_011011611.1 (*P. furiosus*), WP_000197425.1 (*V. cholera*), XP_668452.1 (*C. hominis*), NP_001085699.1 (*X.laevis*), BAD99110.1 (G. gallus), XP_027283074.1 (*C. griseus*), XP_656880.1 (*E. histolytica*), NP_460264.1 (*S. enterica*), WP_013366590.1 (*E. lignolyticus*), WP_053396473.1 (*V. nereis*), XP_003865450.1 (*L. donovani*), XP_001611286.1 (*B. bovis*), CEL76485.1 (*T. gondii*) and XP_688695.4 (*D. rerio*).

Accession No. of Topo IB from various organisms are presented below:

XP_001351663.1 (*P. falciparum*), NP_003277.1 *(H. sapiens)*, NP_014637.1 *(S. cerevisiae)*, XP_034422310.1 (*P. berghei*), XP_001613277.1 (*P. vivax*), XP_725660.1 (*P. yeolii*), XP_740287.2 (*P. chabaudi chabaudi*), XP_628499.1 (*C. parvum*), XP_668245.1 (*C. hominis*), XP_952523.1 (*T. annulata*), NP_990441.1 (*G. gallus*), NP_033434.2 (*M. musculus*), BAN65583.1 (*B. bovis*), XP_002666448.2 (*D. rerio*), NP_001084031.1 (*X. laevis*), NP_001230963.1 (*C. griseus*), XP_019413101.1 (*L. angustifolius*), NP_200342.1 (*A. thaliana*), NP_511161.2 (*D. melanogaster*), NP_596209.1 (*S. pombe*), XP_011393242.1 (*N. crassa*), XP_003436707.1 (*A. gambiae*), AAL38667.1 (*L. donovani*)and EAZ58126.1 (*P. aeruginosa*).

Accession No. of Topo II from various organisms are presented below:

NP_001058.2 (*H. sapiens*), Q6PUA4 (*T. thermophila*), NP_035753.2 (*M. musculus*), NP_001296944.1 (*D. rerio*), NP_990122.3 (*G. gallus*), NP_001082502.1 (*X. laevis*), NP_001233667.1 (*C. griseus*), AAR91745.1 (*E. histolytica*), AAB36610.1 (*S. cerevisiae*), NP_595805.1 (*S. pombe*), Q7Z2D0 (*P. falciparum*), XP_016655535.1 (*P. chabaudi chabaudi*), XP_022813283.1 (*P. yoelii*), XP_001616638.1 (*P. vivax*), XP_034421913.1 (*P. berghei* ANKA),

XP_625680.1 (*C. parvum*), XP_665482.1 (*C. hominis*), XP_953647.1 (*T. annulata*), NP_496536.1 (*C. elegans*), P15348.1 (*D. melanogaster*), NP_001037009.1 (*B. mori*), XP_001611613.1 (*B. bovis*), KAF4639375.1 (*T. gondii*), BAA11510.1 (*D. discoideum*), NP_189031.1 (*A. thaliana*) and AAD34021.1 (*L. donovani*).

Accession No. of Gyrase subunit A from various organisms are presented below:

NP_416734.1 (*E. coli*), XP_001350630.1 (*P. falciparum*), BAP02694.1 (*V. cholerae*), XP_034424206.1 (*P. berghei* ANKA), XP_001617321.1 (*P. vivax*), XP_728165.3 (*P. yoelii*), XP_740732.2 (*P. chabaudi chabaudi*), XP_001609528.1 (*B. bovis*), CEL71871.1 (*T. gondii*), XP_003880046.1 (*N. caninum*), Q7XZF7.2 (*O. sativa*), KOO03512.1 (*V. nereis*), AAS14848.1 (*D. melanogaster*), OAP05190.1 (*A. thaliana*), AAC23563.1 (*S. pneumoniae*), AAA83017.1 (*M. tuberculosis*), NP_461214.1 (*S. enterica*), AAD07753.1 (*H. pylori*) and WP_012855700.1 (*M. hominis*).

Accession No. of Gyrase subunit B from various organisms are presented below:

P0AES6.2 (*E. coli*), XP_001350789.1 (*P. falciparum*), BAP04256.1 (*V. cholerae*), EDO07537.1 (*B. bovis*), CEL72064.1 (*T. gondii*), CEL64794.1 (*N. caninum*), XP_015622284.1 (*O. sativa*), KOO04909.1 (*V. nereis*), AAS13867.1 (*D. melanogaster*), XP_034424367.1 (*P. berghei* ANKA), XP_001613931.1 (*P. vivax*), XP_724272.1 (*P. yoelii*), XP_744228.2 (*P. chabaudi chabaudi),* CAC05495.1 (*A. thaliana*), AAZ93044.1 (*S. pneumoniae*), AAA83016.1 (*M. tuberculosis*), NP_462735.1 (*S. enterica*), AAD07566.1 (*H. pylori*) and WP_012855650.1 (*M. hominis*).

Accession No. of Topo VIB from various organisms are presented below:

NP_188714.2 (*A. thaliana*), J3QMY9.2 (*M. musculus*), XP_001350366.1 (*P. falciparum*), NP_001289013.1 (*H. sapiens*), WP_011012725.1 (*P. furiosus*), VTZ69403.1 (*P. chabaudi chabaudi*), XP_022812488.1 (*P. yoelii*), SCO67967.1 (*P. vivax*), XP_034422620.1 (*P. berghei* ANKA), XP_003543555.1 (*G. max*), B8BDQ4.1 (*O. sativa*), XP_019457683.1 (*L. angustifolius*), XP_005716538.1 (*C. crispus*), O05207.1 (*S. shibatae*), XP_028862395.1 (*P. malariae*), XP_003057307.1 (*M. pusilla*), XP_002177352.1 (*P. tricornutum*), WP_011034341.1 (*M. mazei*), MCC6040583.1 (*D. archaeon*), XP_042753666.1 (*L. sativa*), ETV82550.1 (*A. astaci*) and BAM83247.1 (*C. merolae*).
