# Supplementary material for: A tale of topoisomerases and the knotty genetic material in the backdrop of Plasmodium biology
Source: Biosci Rep. 2022 Jun 14;42(6):BSR20212847. doi: 10.1042/BSR20212847 (PMC9261774; doi:10.1042/BSR20212847)
Supplement: Supplementary Data [file BSR-2021-2847C_supp1.pdf]

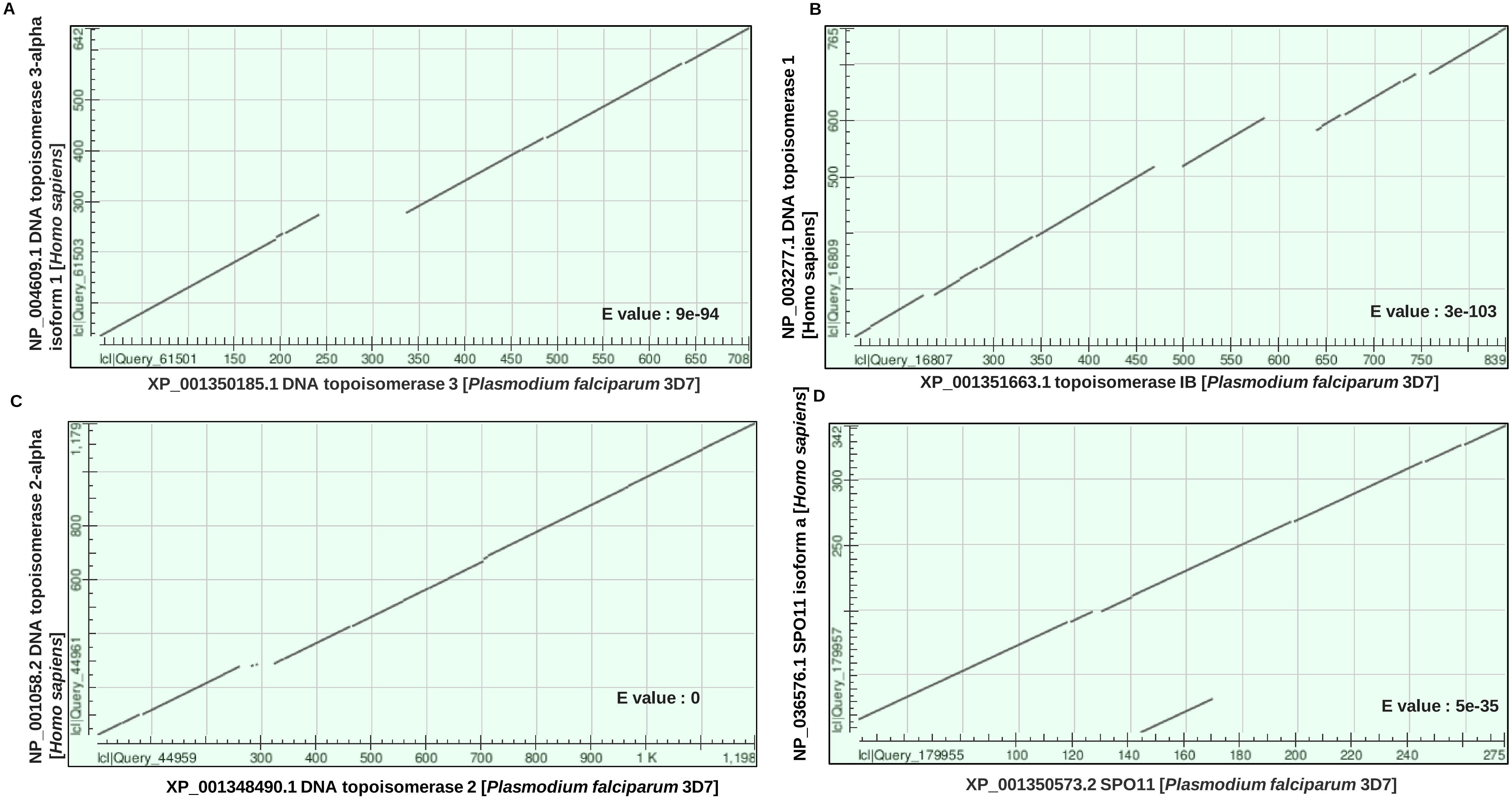

Supplementary Figure S1: Dot Plot showing the sequence comparison between *Plasmodium* topoisomerases with its human ortholog: Each dot matrix represents *Plasmodium falciparum* 3D7 sequence A) *Plasmodium* TopoIII B) *Plasmodium* TopoIB C) *Plasmodium* TopoII and D) *Plasmodium* Spo11 on the X-axis and the sequence of *Homo sapiens* ortholog on the Y-axis. The alignments in the plots are shown as lines. The number of alignments found in BLAST is same as the number of lines present in each plot. The E value for each analysis is presented.
